# Supplementary material for: Are carbon emissions trading and green financial instruments synergistic? -Comprehensive quantitative research based on content analysis
Source: PLoS One. 2024 Mar 7;19(3):e0298601. doi: 10.1371/journal.pone.0298601 (PMC10919601; doi:10.1371/journal.pone.0298601)
Supplement: S1 Data — (ZIP) [file pone.0298601.s001.zip › Data/地级市Z_F工作报告.zip!/░▓╗╒/│n╓▌╩╨/└┤╫╘╣1⁄2╓┌║┼í╛┬φ┐╦╩2╛▌═°í┐_2017.docx]

——2017年1月9日在滁州市第五届人民代表大会第六次会议上

市人民政府市长 张祥安

各位代表：

现在，我代表市人民政府，向大会报告工作，请予审议，并请市政协委员和其他列席人员提出意见。

**一、锐意拼搏向前进，“十三五”实现良好开局**

刚刚过去的一年，是非凡的一年、难忘的一年。面对复杂多变的外部宏观环境，面对艰巨繁重的改革发展稳定任务，面对旱涝频转的自然灾害，面对市委主要领导不断调整的特殊影响，在省委、省政府和市委的坚强领导下，在市人大、市政协的监督和支持下，我们深入贯彻落实习近平总书记系列重要讲话特别是视察安徽、亲临小岗重要讲话精神，坚定信心，沉着应对，主动作为，实现了经济社会“稳中奋进、好中加快”的发展预期，主要指标、主要工作总体上站稳了全省第一方阵。

（一）克服下行压力，主要指标争先进位。保持定力，顶住压力，精准发力，较好实现了换挡不失速、量增质更优的稳健发展。全年完成地区生产总值1423亿元，增长9.2%；规上工业增加值642亿元，增长9.8%；固定资产投资1699亿元，增长16.6%；财政收入256.4亿元，增长11.3%，其中地方收入完成167亿元，增长16.4%；社会消费品零售总额515亿元，增长12.7%；外贸进出口23.3亿美元，增长14.2%；实际利用外商直接投资11.4亿美元，增长8%；城镇、农村居民人均可支配收入分别达到26286元、10956元，同口径均增长8.8%。动态指标中，地区生产总值、固定资产投资、财政收入、社会消费品零售总额、外贸进出口、实际利用外商直接投资、年新增贷款、城镇和农村居民人均可支配收入9项指标增速均居全省前5位。总量指标中，地区生产总值、规上工业增加值、固定资产投资、财政收入、外贸进出口、实际利用外商直接投资、年新增贷款7项指标位居全省第一方阵。

（二）强化四督四保，重点项目加速推进。坚持经济工作项目化、项目工作责任化，大力实施“四督四保”推进机制，一批重大项目进展较快。抓招大引强，新引进亿元以上项目410个，其中近海汽车、长久轿运车、国泰电动汽车、瑞能新能源汽车、沃特玛电池、天鼎丰非织造布、杰事杰新材料等10亿元以上项目超20个。抓项目开工，新开工亿元以上项目480个，华塑二期、中联五期、龙欣新材料、众望科希盟、南方黑芝麻、福莱特玻璃等一批大项目启动建设。抓项目竣工，立讯二期、德威新材料、锦汇新材料、大禹防水材料、欣阳科技、华信玻璃、尚钧医疗等340个亿元以上项目竣工投产。抓项目达产，华汇热电、养元饮品二期、胜华波汽配、天安新材料等104个亿元以上工业项目达产。抓项目转化，滁州产城融合示范区获国家发改委批准，高教科创城首批4个项目开工，西部大工业基地完成规划编研，与南京江北新区合作共建的汊河产业转移承接园正在推进。谋划推进PPP项目，共有27个项目列入国家和省发改委项目库，31个项目列入市级项目库，总投资1200多亿元，已有14个项目与社会资本签约，其中开工建设9个。

（三）着力提质增效，实体经济破难奋进。把发展实体经济放在突出位置，积极落实上级各项决策部署，强化帮扶帮办，把握平衡点，破解困难点，狠抓增长点，经济运行稳中向好。工业经济转型加速。出台多项措施支持企业稳产促销，加快现有产业、企业做大做强。全年净增规上企业124户，总数达1515户，其中产值超亿元企业495户，对全市规模工业贡献率达99%。六大主导产业产值2562亿元，增长11%。战新产业产值639亿元，占规上工业产值比重20.6%，其中省级智能家电基地实现产值411亿元，增长14%，成功创建全国智能家电知名品牌示范区。净增国家级高新技术企业55家，总数达230家，居全省第四；实现产值1436亿元、增加值319亿元，均居全省第三。天长落实稳增长系列措施受到国务院通报表扬，并获批全省首个国家级出口工业产品质量安全示范区；凤阳获批国家循环经济示范县；民企入围全省百强数连续三年居全省第三，蓝德集团进入中国民营企业500强。现代服务业势头强劲。新业态快速成长，建成电商产业园10个、入驻企业近300家；6个阿里巴巴“村淘”县级运营中心、371个村级服务点运营良好，跨境电商实现零的突破，明光获批全国电子商务进农村示范县，云伙计荣获全省十佳产业链服务商，阿里巴巴首个订单农业项目落户定远。定远电商产业园等省级服务业集聚区发展到11家；苏滁产业园、明光医疗产业区跻身长三角首批健康服务产业集聚区，九华药业等4家企业荣获健康服务示范企业；南谯、全椒列入国家全域旅游示范区名录。浦发银行、光大银行滁州分行获批设立、即将开业。现代农业发展加快。全年收购粮食382万吨，居全省第一。现代农业示范区发展到112家，建成省级现代林业示范区4家。新增土地流转面积20万亩，总面积达263万亩，流转率43.4%；新增农业物联网示范点12个，总数居全省第二；新增1家国家级、6家省级农机合作示范社；再添4家国家级、8家省级农民合作示范社和30家省级示范家庭农场。农机化率达81.4%，超全省8个百分点。

（四）坚持精准施策，发展质量明显提高。以推进供给侧结构性改革为主线，加快“调转促”、“全创改”，经济运行质量进一步提升。“去降补”取得新成效。去产能上，压减51万吨水泥产能。去库存上，商品住宅库存面积较上年底减少200万平方米，下降44%，去化周期缩短至6.6个月。去杠杆上，多渠道、多方式扩大直接融资，开润股份成功上市，扬子地板报会待审，新增新三板挂牌企业9家；市城投二期中票成功注册，公司债上交所发行，为全省首家被国家发改委、交易商协会、证交所获准发行直接融资产品的市级平台；设立的10只产业基金投入运营。全年实现直接融资近百亿元，新增贷款222亿元，社会融资总量超500亿元。降成本上，打出“组合拳”，帮助企业减负10亿元以上。补短板上，一批创新平台、基础设施和公共服务等方面项目加快推进。“双创”水平有明显提升。省级创新型试点城市建设扎实推进，全柴动力列入国家技术创新示范企业，金禾、蓝德和扬子空调设立省级博士后科研工作站，院士工作站已建成10家。发明专利授权近1200件，增长85%，均居全省第三。全椒成为国家知识产权强县工程示范县，通用生物获批省级科技创新团队，鸿典科创平台落户市经开区。“天长汇”跻身国家级众创空间，清华大学启迪之星孵化基地、萤火虫创孵梦工厂、天地汇创业孵化器等建成运营，原创科技城启动建设。新登记各类市场主体3.3万户，其中私营企业9000户。

（五）深化改革开放，动力活力加快释放。坚持蹄疾步稳，以解决影响发展的突出障碍、影响民生的突出问题、影响和谐的突出隐患为重点，完成173项年度改革任务。农村综合改革全省领先。土地承包经营权确权登记颁证试点在全省率先完成；天长农村集体资产股份权能改革国家级试点成绩突出，发放全省第一张股权证，率先推出“农权贷”，获农业部肯定；国有林场改革、定远政府购买农业公益性服务、凤阳供销社综合改革、来安“三变”改革等一批国家、省级试点落地生根。放管服改革扎实推进。政府权责清单统一省级试点扎实推进，在全省率先实行“1+X”监管模式；颁发全省第一份“五证合一、一照一码”营业执照、第一份电子营业执照。建成“网上办事大厅”，830项审批服务项目上线运行。创新“互联网+工商服务”模式，实现工商登记注册网上办理。社会事业体制改革再现亮点。医改工作获国家医改办、世界银行肯定，天长率先实施医保“三保合一”、县级公立医院改革成为全国示范，全国医改现场会在天长召开。公车改革深入推进，全国车改现场会来滁观摩；深化户籍制度改革，为2.3万无户口人员办理了户口登记；新型城镇化、国资国企、公共资源交易等改革持续推进。与此同时，对外开放步伐加快，富达电子、同心机电等4家企业在海外投资办厂，累计达21家。天大管材整体出售给法国瓦卢瑞克公司。美大地区友好论坛、光谷跨境电商峰会成功举办。海关天长通关监管中心运行良好，恒欣公用型保税仓库获批。深度融入南京都市圈、合肥都市圈和长三角城市群，全面对接南京江北新区建设，借势借力，取得一批合作成果。

（六）推进城乡统筹，人居环境持续改善。中心城市建设上，全面完成市级“多规合一”，实施重点项目100多个，完成投资50多亿元。聚力推进“四个一”重点工程，琅琊山争创5A级景区步伐加快，北大门建设工程过半，龙池街改造成效初显，南天门建设规划通过审批，农中花源等重点项目崭露新姿；清流河二期主体完工，一期提升、三期规划设计进展顺利；古城保护性改造有序进行，内城河综合治理一期工程完工、二期景观工程快速推进，南大桥、西大街桥通车，清流街、遵阳街改造启动；明湖完成24平方公里控规和城市设计工作，大坝工程、湖区土方工程等快速推进。龙蟠大道下穿铁路、西涧北路、天长路改造、紫薇北路、醉亭路等基本完工，金叶路、新安江路等加快施工。15个居民小区二次供水改造全面推进。加快建设智慧城市，推行网格管理，争创省级文明城市，推动城市管理上台阶、上水平。县城和镇村建设上，6个县城实施重点项目380个，老城改造有序推进，新区拓展方兴未艾，园区提升步伐加快，县城建设中的精品力作不断呈现。乌衣、汊河、炉桥、临淮、秦栏、大墅等重点镇加快建设。美丽乡村建设由“以点为主”向“由点到面”转变，开展“百镇治理”，分层推进中心村建设。在天长全域开展美丽乡村建设试点，启动“小岗三年大提升”行动，来安贾龙村入选全国少数民族特色村。天长和定远分获沿江片、皖北片美丽乡村建设先进县。全省农村生活垃圾治理现场会在全椒召开。基础设施建设上，宿扬高速基本建成，滁淮高速完工近半，合宁“四改八”开工，来六、滁天、明巢、滁城西环4条高速加快前期工作，滁宁轻轨完成工可编制，滁州机场初步选定3处场址。滁州港（汊河）开港，滁河防洪治理近期工程完工，淮干蚌浮段行蓄洪区调整、池河治理等工程加快实施，江巷水库开工建设。农村道路畅通工程建成2800多公里，居全省前列。

（七）突出民生保障，群众获得感不断提升。持续实施33项民生工程，全年民生支出290.5亿元，占财政支出的86.8%。新增城镇就业8.2万人，超目标任务一倍多。城镇登记失业率3.2%，低于省控目标1.3个百分点。新开工保障性住房1.1万套，建成1.7万套，货币化安置6600多户，发放公积金贷款24亿元。农村危房改造开工率、竣工率均达113%，居全省前列。精准扶贫、精准脱贫首战告捷，经过省脱贫攻坚第三方监测评估，5个重点县顺利实现4.8万人脱贫、55个村出列，超额完成省下达的年度任务；定远金融扶贫成为全省样板，在全国作经验交流。坚持防汛抗旱两手抓，夺取抗洪救灾全面胜利。社会事业发展迅速，学前教育二期三年行动计划全面完成，在全省率先开展普惠性民办幼儿园认定工作，加快推进城区10所中小学整合扩建。全椒、来安、定远、凤阳和明光义务教育均衡发展通过国家验收。城市职业学院新校区正式启用，新技师学院整合成立。市科技馆开馆，市博物馆完成布展，市图书馆主体封顶，长城影视成为省级影视拍摄基地。市一院南区投入使用，南京儿童医院滁州分院二期加快建设。市福利中心扩建工程一期完工。成功举办首届国际半程马拉松赛、全国百城自行车赛、中国农民歌会第二季滁州演唱会等。道德信贷工程在全国推广，安徽好人、中国好人上榜人数居全省前列。深化军民融合发展，荣膺全国双拥模范城“五连冠”、全国人民防空先进城市等称号。防震减灾工作跻身全国前十。推进绿色发展，完成造林18万亩、城镇绿化800万平方米，百万亩森林增长工程圆满收官。西涧湖二级保护区整治成为全国样板。顺利完成PM10年度控制目标和主要污染物减排任务，秸秆禁烧实现重点时段“零火点”。突出重点行业领域安全监管，事故起数和死亡人数连续五年双下降。食品药品安全工作得力、形势较好。深化司法体制改革，推行阳光信访，扎实开展平安滁州建设，和谐稳定局面进一步巩固。红十字会、慈善和残疾人等事业取得新成绩，工会、共青团、妇联、文联、科协等群团组织作用进一步发挥，外事侨务、民族宗教、编制、物价、对台、保密、档案、地方志、工商联、关心下一代等工作全面进步。

十件实事取得成效。滁州农产品批发市场投入运营，农贸市场迁建改造有序推进；棚户区和老旧小区年度改造任务超额完成；市植物园完成前期准备；南谯路畅通工程正在实施；城北客运站主体工程完工；出台《滁城限制养犬管理办法》，规范了养犬行为；老旧电梯维护更新力度加大，在全省率先建成应急救援平台；第三污水处理厂工程加快实施；滁宁公交换乘中心建成运营；新增公交车36辆，增建公共自行车站点31个。

（八）严格依法行政，自身建设得到加强。扎实开展“两学一做”学习教育和“讲看齐、见行动”学习讨论，坚持政府常务会议学法和重要文件集体学习解读制度。自觉接受人大法律监督、工作监督和政协民主监督，办理人大议案4件、建议104件、政协提案398件，满意和基本满意率超过98%。启动政府立法，提请市人大常委会审议法规案2件、制定政府规章1部，其中《滁州市市区饮用水水源保护条例》颁布实施。落实重大行政决策程序制度，推进合法性审查机制全覆盖，开展行政执法公示试点，提升依法执政水平。持之以恒落实中央八项规定和省、市委有关规定，驰而不息抓作风、纠“四风”。加大政务信息公开力度，开通政务微博微信，提高市长热线服务水平。推进网络问政和阳光政务，市本级和明光政务服务中心被列为国家公共服务综合标准化试点。全面实施财政预决算和“三公”经费公开，全市“三公”经费支出下降7.5%。落实“两个责任”，履行“一岗双责”，深入开展政府系统党风廉洁建设和反腐败斗争，加大正风肃纪力度，强化行政监察和审计监督，努力从源头上预防和治理腐败。

各位代表！在经济下行压力加大、困难挑战增多的情况下，去年取得的成绩实属不易。这是省委、省政府高度重视、亲切关怀的结果，是市委坚强领导、科学决策的结果，是市人大、市政协和社会各界关心支持、倾力帮助的结果，更是全市人民迎难而上、奋力拼搏的结果。在此，我代表市人民政府，向全市人民，向人大代表、政协委员和党代会代表，向各民主党派、工商联、无党派人士和各人民团体，向中央及省驻滁单位，向人民解放军、武警官兵、公安政法干警，向所有关心、支持、参与滁州建设与发展的各位老领导、老同志和各位客商、各界朋友，表示衷心的感谢，并致以崇高的敬意!

在肯定成绩的同时，我们也深刻认识到工作中还存在不少困难和不足，主要是：经济下行压力较大，新的增长动力不足，部分企业生产经营困难，个别指标增速没有达到预期目标；一些重点领域和关键环节的改革突破不够；新的大项目偏少，创新能力较弱，转型升级任重道远，资源环境等瓶颈制约加剧；民生问题仍有不少欠账，脱贫攻坚任务艰巨，教育、医疗、住房、养老等领域的供需矛盾仍很突出；少数政府部门和工作人员主动服务和勇于担当意识不强，文多会多检查多问题回潮，等等。对此，我们一定要高度重视，加强整改，努力在实践中把各项工作做得更好。

**二、撸起袖子加油干，坚决完成今年各项目标任务**

今年是党的十九大胜利召开之年，是实施“十三五”规划的重要一年，是供给侧结构性改革的深化之年，也是本届政府任期的最后一年。做好今年工作，意义重大。

今年工作的指导思想是：全面贯彻党的十八大和十八届三中、四中、五中、六中全会精神，深入贯彻习近平总书记系列重要讲话特别是视察安徽重要讲话精神，认真落实中央经济工作会议、省第十次党代会和市第六次党代会部署，聚焦“346”奋斗目标，以五大发展理念为统领，以供给侧结构性改革为主线，以提高发展质量效益为中心，稳中奋进、好中加快、能快则快，加速冲刺总量全省第三，以优异成绩迎接党的十九大胜利召开！

经济社会发展主要预期目标是：地区生产总值增长10%，规上工业增加值增长11%以上，固定资产投资增长15%以上，财政收入和城乡居民人均可支配收入与地区生产总值同步增长，社会消费品零售总额增长12%左右，城镇登记失业率控制在4.4%以内，完成节能减排任务。

确定10%的增长目标，是市委、市政府广泛听取意见、综合权衡分析后决定的。确定这一目标是必要的。我们要实现“四个翻番”、冲刺总量全省第三的奋斗目标，GDP必须保持10%的年均增长水平，前年和去年已有欠账，越往后基数越大，若今年再有欠账，后几年要如期实现这一目标，就会十分困难。实现这一目标是可能的。今年我市的重大基础设施项目较多，房地产形势会进一步趋好，与此关联的建筑业将会有更好表现，前几年新上的工业项目陆续投产达产，服务业支撑经济发展的动力强劲。今年是党的十九大胜利召开之年，经济发展的政策环境相对宽松，利好因素相对增多。关键是我们能否把握机遇，因势利导，奋力拼搏，保持冲刺上行的良好态势，实现经济社会更好更快发展。

重点做好九个方面工作：

（一）更加注重创新创业，鼎力培育发展动能。强化需求导向，在新技术、新产品、新模式、新业态、新政策以及新体制机制等方面不断创新，转换发展动能，厚植发展优势。

深入推进“去降补”。落实“1＋7”方案，进一步优化存量、引导增量、主动减量，推动供需两端协同发力。优化结构去产能，鼓励企业通过主动压减、兼并重组、转型转产等途径，积极化解过剩产能，促进水泥、玻璃行业结构优化、提质增效。因城施策去库存，坚持“房子是用来住的、不是用来炒的”定位，综合运用各种调控手段，合理出让居住用地，推进公租房改革和棚改货币化安置，加快消化非住宅商品房，依法加强商品房销售监管，适时调整住房公积金贷款政策，保持房地产市场平稳健康发展。全年完成房地产开发投资300亿元。积极稳妥去杠杆，不断扩大直接融资规模，鼓励更多企业开展股权融资和企业上市挂牌，引导商业银行对市场前景好、暂时有困难的企业不断贷、不抽贷，推动已上市挂牌公司通过再融资、并购重组等方式做大做强。力争全年新增首发上市企业1家、报会待审2家、辅导备案3家、“新三板”挂牌10家以上，上市企业后备库动态保持在150家以上，完成直接融资超100亿元，新增贷款超230亿元。积极防范金融风险和地方政府债务风险，严控政府债务规模，加快存量债务置换。多措并举降成本，进一步降低企业税费、融资、交易、物流、能源、财务等成本，减轻企业负担，提高企业效益。突出重点补短板，在创新能力提升、新兴产业培育、基础设施建设、公共服务配套等领域，实施一批补短板重点项目。加强政策引导，进一步完善土地、财政资金等要素配置机制，广泛引入社会资本，增强公共产品供给能力。

大力实施创新驱动战略。坚持把创新作为第一动力，在更大范围、更广领域、更深层次推动创新，精心打造经济社会发展核心引擎。一是培育创新主体。年净增高新技术企业40家以上、企业研究院等研发平台2家以上、授权发明专利1400件以上。各县市区和市经开区、苏滁园至少与2家国家级一流院所紧密合作；每个亿元以上企业至少有1家技术依托单位，有1个以上新技术或新产品研发创新团队，鼓励企业培育、引进多层次的行业工匠。二是完善创新体系。围绕“三重一创”，打造一批重点产业基地，建设一批重点产业工程，实施一批重点产业专项，加快构建创新型现代产业体系。围绕高水平研发平台建设、高层次团队引进、高科技项目争取等方面，制定出台科技创新相关政策，进一步健全完善人才、金融、服务等政策体系，激发创新活力。三是搭建创新平台。整合提升省级高新技术园区和产业集聚区，加快高教科创城、原创科技城、中国家电研究院安徽分院和国家电子元器件质检中心等平台建设。院士工作站力争再翻一番。四是集聚创新人才。坚持培养和引进并重，更加注重柔性引进、项目引进、专项资助引进，实施高层次人才引进计划，开展引博、院士助滁行动，充分发挥鸿典集团等平台集聚专家团队的作用，力争在引进产业人才、工科类工程类院士上有新突破。

加快推进大众创业。加大对高校毕业生、农村转移劳动力等重点群体创业的服务保障，完善扶持政策，优化创业环境。积极发展众创、众包、众扶、众筹，力争新增市级以上众创空间8—10个。加快民营经济发展，鼓励企业家“二次创业”，引导在外成功人士返乡创业，让市场活力充分涌动。新增个体工商户1.8万户、私营企业5000家以上，民营经济增加值增长11%。

（二）更加注重难点重点，着力深化改革开放。禀承滁州人的改革基因，弘扬大包干的改革精神，紧扣“四个有利于”的改革方向，坚持系统思考谋划，突出问题导向，革故鼎新，继续拓展对外开放的广度、深度，不断增创发展动力、活力。

进一步深化重点改革。农村综合改革上，坚持“抓小岗带全市”，重点在“三权分置”和“三变”改革等方面寻求突破。推进颁证后续工作，强化确权成果利用。以集体资产股份权能改革为突破口，加快推进农村产权制度改革。鼓励支持农村金融创新，加强农村产权流转交易市场建设，扩大农村“三权”抵押贷款试点。推动政府购买农业公益性服务常态化。全面推开农业“三项补贴”改革。完成35个国有林场改革，深化供销社综合改革、小型水利工程管理体制改革等。“放管服”改革上，依托“互联网＋政务服务”，持续推进行政审批流程再造。健全“3+2”清单制度体系，扎实推进全国政务服务标准化试点。深化商事制度改革，推进企业登记全程电子化，加强事中事后监管。推进公共资源交易监管体制改革，健全市县一体化公共资源交易平台。财税金融改革上，深化预算管理制度改革，推进国、地税征管体制改革。完善“产业+基金”运行模式，用足用活产业发展基金。持续推进“4321”新型政银担试点，完善政策性融资担保风险补偿机制。新型城镇化改革上，以滁城、各县城和乌衣、汊河、炉桥等地为重点，以深化户籍制度改革为切入点，切实抓好进城人员“三权落实、五有并轨”，力促人口向城镇集聚。围绕农民市民化、投资多元化、管理规范化、建设特色化，走出具有滁州特色的新型城镇化之路。社会事业体制改革上，扭住“三医联动”“三保合一”“招采合一”等关键环节，巩固扩大医改成果，力争在全省乃至全国再添新亮点。积极探索在教育、医疗领域实行“编制周转池”使用方式改革。开展市直单位用人清理、规范专项行动。继续推进社保、文化等领域改革，不断增强群众对改革成果的获得感。

进一步扩大区域合作。顺应国家“一带一路”、长江经济带战略，发挥比较优势，坚持引进来与走出去相结合，力争全年新增进出口实绩企业100家以上，进出口总额有明显增长。深度融入长三角，加快产业、交通、市场、通关及公共服务等一体化进程，推动我市与苏浙沪联动发展。抓住省里将滁州打造成省际交汇区域中心城市的机遇，对毗邻南京特别是江北新区区域进行系统规划，主动对接、等高对接、无缝对接，推动整体融合、功能互补、错位发展，全力建好滁州产城融合示范区；抓住省里支持滁州加快与合肥一体化步伐的机遇，深化两地在基础设施、产业布局、要素市场等方面合作，进一步构筑“双圈互动、左右逢源”的区域发展新格局。落实省促进皖北发展政策，积极参与淮河流域治理和淮河生态经济带建设，促进沿淮区域加快发展。

（三）更加注重转型升级，倾力壮大实体经济。坚持扩量与提质、特色与创新并举，着力打造现代产业体系，提高产业整体实力和竞争力。

矢志不渝推进工业强市。按照总量扩大、结构优化、产业升级的要求，加快提升工业发展水平。大力培育新兴产业，抓好省级智能家电基地建设、市级战新产业基地培育，力争战新产业实现产值700亿元以上。加快改造传统产业，分行业研究传统产业提升的主攻方向和政策措施，突出产业配套、链条延伸、集聚发展，加快建设重点产业集群。实施“千亿技改”“千企升级”，落实5000万元以上技改项目300个，力争全年实现工业投资1000亿元，其中技改投资420亿元，六大主导产业产值2770亿元。鼓励扶持企业争创名牌名标，大力提升品牌竞争力，全年新增省级以上名牌名标34个。提高产品本地配套率，力争博西华、康佳电子、猎豹汽车、全柴、扬子空调等5家企业本地配套率提升2—8个百分点。实施分类指导，实现帮扶工作常态化、精准化、责任化。切实尊重、关爱企业家和企业经营者，充分发挥他们在振兴工业中的主体、主导作用。

千方百计发展现代服务业。着力打造“电商滁州”。加快市电子商务产业园、孵化中心等建设，扶持云伙计等加快发展，推动车团网等加快落地。加强与阿里、京东、苏宁等知名电商合作，推动产销企业强化线上线下合作，扶持“村淘”等涉农平台发展。实施电商培育计划，孵化电商企业100家、培训从业人员1万人。以更快的步伐推进旅游业发展。大力发展旅游+生态、旅游+文化、旅游+体育，打造以休闲娱乐、观光游览、文化体验等为主题的精品旅游线路，推进“赛事兴旅”。大力发展研发设计、金融服务、现代物流等生产性服务业。力争服务业增加值增长11.5%。

多措并举提升农业发展水平。把农业放在一二三产融合发展的大格局、大体系中来思考和谋划，更加重农强农，扎实推进农业供给侧结构性改革。加快农业产业化，进一步加大政策倾斜力度，加快农业园区建设，培育发展新型农业经营主体，构建立体式现代农业经营体系。力争新增市级以上农业示范区10家、家庭农场1000家。优化种养结构，因地制宜招引农副产品精深加工企业，一对一组织现有食品企业去县市区共建原料基地，把田间变为工业第一车间，提高农副产品就地转化率。推进生产机械化，运用机械化装备和信息化技术，提高现代化种养水平，突出解决粮食烘干装备不足问题，推广耕地质量建档立卡、测土配方施肥技术，提升农业综合生产能力和效益。加速营销电商化，大力推进电子商务进农村，既解决农产品卖出问题，又促进农民扩大消费，兴旺农村市场。推广模式复合化，推进农业与旅游、休闲、体验、健康、文化等有机融合，使其功能叠加、价值增加，开辟现代农业发展新的蓝海。

因地制宜提振县域经济。把县域经济作为全市加快发展的基石，围绕做大规模、做强产业、做出特色，制定县域经济发展推进计划，促进县域分工协作，实现特色发展、错位发展、竞相发展。完善督查考核办法，力促各县市区在全省考核中增速争先进、总量进位次、人均上台阶。支持天长招大引强和育大培强，加快新型工业化步伐，提升全省十强县位次，尽快挤入全国百强县，进一步走在前、作表率；加快来安、全椒市域副中心城市建设步伐，推进滁来全协同发展、板块联动；推动明光、定远、凤阳联手打造西部大工业基地，搞好规划引领，优化资源配置，强化龙头带动，进一步上规模、促转型；鼓励南谯、琅琊大力发展现代服务业和都市农业，进一步扩总量、快发展。支持县域工业园区创新发展，提升开发水平，打造支撑县域发展的增长极。

（四）更加注重效率效果，强力抓好项目工作。项目是拉动发展的重要载体，是衡量发展的具体验证，必须认真落实“四督四保”要求，始终如一、全力以赴抓紧抓好项目工作。

狠抓招商引资。坚持把招商引资作为经济工作的头号工程、第一要务，多措并举、多点发力、多向突破，以更大决心、更大力度加以推进。突出精准招商。由过去的粗放式向精细化、专业化、专题化转型，以商招商、专业招商、产业链招商并举，高位推进，引资引智引技引团队，进一步提升招商引资成效。突出招大引强。集中人力、财力、精力，攻坚大项目，力争在引进50亿、超百亿的龙头型、旗舰型、基地型项目上取得突破。突出“招”“安”结合。进一步完善项目审批“绿色通道”，按照“零障碍、低成本、高效率”的要求，实行跟踪服务、上门服务和全程服务。切实加强土地、劳动力、资本等要素保障，着力解决外来客商的居住环境、子女入学、医疗卫生等问题，让投资者安心创业、舒心生活。突出队伍建设。加强招商人员培训，尽快培育一批具备国际视野、熟悉国际规则的专业化人才。充分发挥好第二批县干招商小组作用。力争引进市外亿元以上项目400个，到位资金900亿元。

加快项目推进。紧紧扭住项目这个牛鼻子，以亿元以上项目为重点，层层压实责任、传导压力，进一步提高项目开工率、竣工率、达产率和转化率。开工建设上海电缆、扬子新能源汽车、九次方健康医疗大数据、寒锐钴业等项目400个；加快推进拉芳家化、量子光电、德青源食品、椰牛食品等400个在建项目；力促博西华洗碗机、焕发生物、兴扬特种车、鸿耀物流园、博成包装等300个项目竣工；谋划储备项目600个以上，力求某薄膜太阳能、某TFT新型显示、某纯电动汽车、某光刻胶、某饮料、某豆奶、某电视机等项目签约落地，促成更多项目纳入国家和省项目库。

提升园区平台。坚持产城融合理念，推进各类开发区转型升级，促进园区生产、生活、生态空间有机协调。进一步加大投入，提高配套水平和承载能力。市经开区要勇挑重担，创新发展，争取总量超千亿。苏滁产业园要突出招大引强，深化与中新集团合作，确保全年新签约、新开工、新投产亿元以上工业项目分别达20个、15个、10个以上，其中引进30亿元工业项目1个以上。加快建设创新型园区，力争市经开区、苏滁产业园高新技术产值占比达65%以上，其他园区达50%以上。推动8个筹建省级开发园区“去筹转正”、力争2个以上获批，争取天长开发区成为国家级高新区、全椒开发区扩区获批，推进凤阳·宁国、定远·肥东、明光·肥西合作共建园区。

（五）更加注重建管一体，合力推进城乡建设。以人的城镇化为核心，顺应发展新趋势，加强城镇规划建设管理，着力打造宜居宜业的城乡环境。

提升规划水平。加大新版滁州城市总规执行力度，完善近期建设规划，推行“多规合一”。把以人为本、尊重自然、传承历史、绿色低碳等理念融入城市规划全过程，注重留白、留绿、留旧、留文，展现“山城相依、水城交融”的山水亭城特色。按照生产空间集约高效、生活空间宜居舒适、生态空间山青水秀的要求，全面开展城市设计，让每个城镇都有独特的历史文化符号。

加快城乡建设。大滁城建设上，紧紧围绕“一主四副一依靠”，背依琅琊山，建好主城区，打造好全椒县城、来安县城和南谯新城、汊河新城四个副中心，在提升内部同城化的同时，呈扇面状向南京推进，全面对接南京。深入推进“四个一”工程，力争琅琊山创建5A级景区取得实质进展；清流河综合治理三期、四期全面启动；古城依托内城河改造、遵阳街改造、北湖治理等，尽快展现古城风韵；明湖大坝确保今年汛前下闸蓄水，开工建设景观工程和路网工程，力争开工建设奥体中心等。城南新区着力完善功能配套，推进学校、医院、菜场及邻里中心等建设，尽快形成功能齐全、人气旺盛的城市新组团。以原创科技城为抓手，加速高铁站区开发步伐。建成琅狮大桥，打通城南新区与苏滁产业园的快速通道。老城区实施南谯北路改造，打通紫薇路，北延中都大道。启动三水厂二期扩建工程，建成第三污水处理厂。城北客运站正式启用。城镇建设上，天长、明光、凤阳、定远4个县城要坚持产城结合、建管结合、新老结合、城乡结合，加快扩容提质，进一步完善功能、强化管理、提升水平。因地制宜、突出特色，大力推进重点镇建设，支持秦栏、水口、炉桥、大墅等建设特色小镇，构建多点支撑的城镇体系。全面完成“百镇计划”“基本版”和50%乡镇“升级版”建设任务。美丽乡村建设上，统筹规划、精心安排40个左右省市级中心村建设，注重保护好自然生态、田园风光，守住历史风貌、乡土气息，力戒乡村城市化，力避新村空心化。

强化规范管理。以创建全国文明城市、国家森林城市、环保模范城市为抓手，全面提升城乡管理水平。推进城市执法体制改革，依法构建权责清晰、服务为先、管理优化、执法规范的新机制。主攻重点，提升网格化、常态化等“七化”水平，加强渣土运输、排档摊点、户外广告、城市养犬等管理。推进社会服务管理信息化建设，整合数字城管、平安城市等系统，构建智慧城市综合管理体系。围绕群众关心的交通拥堵、物业管理、车辆停放等问题，下硬手、出实招，有效缓解“城市病”。大力推广垃圾分类试点和清洁收运，加快垃圾减量化、资源化、无害化处理。

完善基础设施。把滁宁城际铁路作为今年的“一号工程”，力争年内开工建设。加速推进滁淮高速、合宁高速“四改八”，开工建设滁六、滁天、明巢、滁城西环4条高速，加快合青高铁、合滁宁高铁、宁淮客专、滁州机场等前期准备，抓好滁州大道、洪武西路等12条干线公路建设，完成农村道路畅通工程2460公里。完善凤阳港功能，推进全椒、明光新港建设。加快江巷水库、淮干蚌浮段行蓄洪区调整工程建设，实施黄栗树水库、沙河水库除险加固及增蓄工程，推进山许水库和滁河防洪（新增）工程前期工作，抓好小流域治理、小水库除险加固及清淤、农村饮水安全、八小水利等工程。新建通信基站1500个，完成投资15亿元。加快电网建设，完成220千伏定远韭山变、110千伏天长沃公变等。力争中联能源热电联产项目投产，中海油滨海液化天然气配套管线滁州段项目开工建设，推进大唐电厂、中海油滁州燃气电厂项目，加快城市供气、供热设施建设。

（六）更加注重保护治理，奋力守护绿色家园。牢固树立绿水青山就是金山银山的理念，切实保护好、治理好、利用好生态环境，促进人与自然和谐共生。

实行铁腕治污，让美丽家园更净。顺应群众对清新空气的期待，坚持源头严防、过程严管、后果严惩，持续开展大气污染治理。建立秸秆禁烧和综合利用常态化机制，继续加强烟花爆竹限放管理。主城区全面取缔燃煤小锅炉、淘汰黄标车。进一步加强建筑扬尘治理，力争滁城建筑施工扬尘治理率达100%。严格落实“河长制”，巩固滁河陈浅等4个、改善池河公路桥等3个国省考核断面水质。构建土壤环境监测和生物多样性保护网络，强化农业面源污染防治和工业污染场地治理。全面提升环评审批、总量减排、环境监测等水平，加大环境执法力度，解决突出环境问题。

加强生态建设，让皖东大地更绿。大力实施“五森工程”，完成造林18.5万亩。深入开展“两型”示范创建，推进生态市县、美丽乡村、绿色社区等试点示范建设，完成42个镇污水处理项目建设，力争省级以上生态乡镇占比达50%。深入实施山水田湖生态保护和修复工程，加快饮用水源地环境综合治理，推进琅琊山山体生态修复，抓好水生态文明城市建设试点，开展清洁小流域和坡耕地水土流失整治。

突出集约节约，让资源利用更好。积极开展全民节约行动，推广绿色节能建筑，发展低碳绿色公共照明，推进低碳交通发展，实施新能源汽车推广计划。发展循环经济，推动废旧汽车、电子家电、电线电缆等“城市矿产”资源规模化、规范化和高值化利用。力争大唐张山、中广核猫头山风电项目及凯迪全椒、国祯明光秸秆电厂开工建设，建成光大南谯等3个项目，推进天长生物质发电项目。加快矿产资源整合，做大做强兴滁矿业，最大限度发挥效益。实行最严格的节约用地、最严格的水资源管理和最严格的能耗强度控制制度，推进资源节约利用。

（七）更加注重协调协同，努力繁荣社会事业。进一步处理好经济发展与社会进步的关系，大力发展各项事业，提供更公平更优质更全面的公共服务。

优先发展教育事业。进一步优化布局、提升质量，全力抓好学前教育、义务教育和高中教育，加快现代职教体系建设，发展优质民办教育，构建多元化办学格局。实施乡村教师支持计划。启动明湖中学建设。支持安徽科技学院、滁州学院加快发展。推进职业技术学院创建地方技能型高水平大学、城市职业学院创建地方特色型大学。发展特殊教育，谋划建设特教学校。

大力发展文化事业。践行社会主义核心价值观，深入开展精神文明创建活动，注重家庭家教家风，提高网络管理水平，弘扬正能量。实施文化惠民工程，完善公共文化服务网络，提高基层文化服务功能。加快市图书馆建设，完成10个省级农民文化乐园、10个市民文化乐园、10个乡镇综合文化服务中心建设任务。加大文物古迹和非物质文化遗产保护力度。积极谋划一批能够吸引社会投资、带动作用强、市场前景好的重大文化产业项目，深度发掘明文化、醉翁文化、儒林文化等地域文化内涵，推进长城文创、农中花源等项目建设。完成章益、吴棠等名人故居修缮工作，打造历史文化街区，争创省级历史文化名城。推进广播电视村村通向户户通升级。加强对外宣传，传播滁州声音，讲好滁州故事，打造“江淮领秀、醉美滁州”的城市名片。

加快发展卫生健康事业。牢固树立“大健康”理念，推动卫生与健康工作由“以治病为中心”向“以人民健康为中心”转变，从注重“治已病”向“治未病”转变，打造健康滁州。做精做强市一院、中西医结合医院，推进市传染病、精神病院建设。加快培养全科医生，加强乡村医生队伍建设。广泛开展全民健身活动，大力推进公共体育设施建设。坚持计划生育“三个不动摇”，提升出生人口素质。高度重视“舌尖上的安全”，积极争创食品药品安全市。

统筹做好各项工作。推动军民深度融合发展，深化双拥共建，扎实做好优抚安置工作，建成市国防动员训练基地二期工程。推进皖东强对流天气监测预警中心建设，提高气象防灾减灾能力。支持工会、共青团、妇联、文联、科协等群团工作，进一步加强外事、侨务、宗教、统计、物价、质量、编制、档案、保密、人防、地震、消防、老干部、地方志、红十字会和应急管理等工作。

（八）更加注重共建共享，全力增进民生福祉。坚守底线、突出重点、完善制度、引导预期，切实保障和改善民生，不断提高市民的获得感、幸福感。

突出打好脱贫攻坚战。把脱贫攻坚作为重大政治任务和最大民生工程，按照精准扶贫、监测评估、防范返贫、责任落实“四个全覆盖”要求，以产业扶贫为根本，以健康扶贫为重点，深入实施脱贫攻坚十大工程。推进台账管理，强化节点管控，落细落准落实攻坚任务，全年完成30个以上村出列、4万人脱贫。完善产业政策支持机制，大力实施带动性强的产业扶贫项目，着力增强内生动力。创新贫困人口医疗卫生服务体制机制，解决好因病致贫、因病返贫问题。

切实强化民生保障。完成33项民生工程年度任务。大力实施“创业皖东”“技能皖东”“就业扶持”工程，统筹做好重点群体人员就业工作，新增城镇就业4万人，确保零就业家庭动态“清零”。全面落实机关事业单位养老保险制度。重视发展养生养老、医疗保健产业，满足多层次、多元化的养老康体需求，构建居家为基础、社区为依托、机构为补充、医养相结合的养老服务体系。健全社会救助体系，深化救急难全国试点，加大困难群众救助力度。完善农村留守儿童和老人、残疾人关爱服务体系，支持慈善、社会福利事业发展。

不断创新社会治理。加强人民调解与行政调解、司法调解衔接配合，构建多元化矛盾化解体系。落实信访工作责任制，推进法治信访、阳光信访和责任信访，完善“乡为主”机制，及时就地解决群众合理合法信访诉求。深化平安滁州建设，加强社会治安综合治理，坚决防止发生重大群体性事件，确保群众安全感满意度“双提升”。严格落实安全生产责任制，加强重点行业、重点领域专项整治，深入推进“铸安”行动，严管重罚，坚决遏制重特大事故发生。

着力办好为民实事。市政府今年继续为市民办好十件实事，包括：改造滁城棚户区2000户；完成4个老旧小区改造和20个安置小区配套工程；建成街头游园10个；新建城南新区公厕5座；完成10个居民小区二次供水改造工程；实施4个社区公共服务用房达标工程；实施10处人行道、盲道完善工程；建成8处流动摊点集中便民服务点；实施8条城市主干路亮化复绿工程；迁建市花鸟鱼虫市场。

（九）更加注重从严从实，致力政府自身建设。认真践行“三严三实”，以忠诚为魂、担当为要、落实为本、法治为纲、廉洁为基，持之以恒加强政府自身建设。

不忘初心永葆忠诚。始终把对党绝对忠诚作为干事创业的灵魂，深入贯彻习近平总书记系列重要讲话特别是视察安徽重要讲话精神，严格遵守《党章》，认真执行《关于新形势下党内政治生活的若干准则》和《中国共产党党内监督条例》，深入开展“讲看齐、见行动”学习讨论。进一步树牢“四个意识”，尤其是核心意识、看齐意识，始终在思想上政治上行动上同以习近平同志为核心的党中央保持高度一致，坚定不移向党中央看齐，向党的理论和路线方针政策看齐，向党中央决策部署看齐。始终把纪律和规矩挺在前面，以身作则、以上率下，统一步调、令行禁止，层层立标杆、作示范。

不辱使命敢于担当。始终把敢于担当作为思想自觉和行动自觉，不能只想他人担当、自己不担当，不能嘴上说担当、遇事不担当，不能合意的就担当、不合意的不担当。反对推诿扯皮、反对久拖不决、反对畏难却步、反对投机取巧、反对粗枝大叶、反对虎头蛇尾、反对揽功诿过、反对评头论足。落实“三个区分开来”，实化、细化容错纠错免责机制，真正做到为担当者担当、为负责者负责，营造更加公正、更加智慧、更加理性、更加包容的政治生态。创新激励机制，对干部政治上激励、工作上支持、待遇上保障、心理上关怀，让广大干部安心、安身、乐活、乐业。

不折不扣狠抓落实。始终把抓落实作为政府工作的主旋律，发扬“钉钉子”精神，对看准的事、定下来的事、干起来的事，舍得下真功夫、花大气力，以踏石留印、抓铁有痕的劲头抓下去，保持力度、保持韧劲，紧抓不放、一抓到底，善始善终、善作善成。完善目标考核，加强政务督查和效能建设，多说“Yes”少说“No”，只说“Yes”不说“No”，推行马上就办、办就办好。进一步精简会议文件、改进会风文风，倡导务实作风，身体力行，靠前指挥，出实招、重实干、亮实绩。

不留盲区接受监督。始终把依法行政作为工作准则，履行好党政主要负责人推进法治建设第一责任人职责，加快法治政府建设。自觉接受人大、政协和纪检、司法、社会各界监督，主动听取民主党派、工商联、无党派人士和人民团体意见建议。认真办理人大议案、建议和政协提案。推进依法科学民主决策，提高政府立法质量，加强规范性文件审查管理。强化执法监督，推进“双随机一公开”，做到严格规范公正文明执法。加强政务信息公开、舆情处理回应和政策研习解读，推进政务微博微信建设，让权力在阳光下运行。

不遗余力从严治政。始终把廉洁从政作为政府工作的底线，坚定不移把反腐倡廉推向深入。严格落实全面从严治党主体责任和“一岗双责”，认真执行《中国共产党廉洁自律准则》《中国共产党纪律处分条例》，严格遵守中央和省委、市委各项规定，持之以恒反“四风”。织密扎紧“制度笼子”，切实管好公务人员、公共资源、公共权力、公共资金。加强行政监察和审计监督，坚决纠正不作为、乱作为，坚决克服懒政、怠政，坚决惩处失职、渎职，切实做到为民务实清廉。

各位代表！滁州加快发展其时已至，干事创业其势已成。让我们紧密团结在以习近平同志为核心的党中央周围，在省委、省政府和市委的坚强领导下，抢抓机遇，乘势而上，砥砺前行，奋勇争先，不断开创“冲刺总量全省第三、同步实现全面小康”的崭新局面，以优异成绩迎接党的十九大胜利召开！
